# Supplementary figures and images for: The dark kinase STK32A regulates hair cell planar polarity opposite of EMX2 in the developing mouse inner ear
Source: eLife. 2023 May 5;12:e84910. doi: 10.7554/eLife.84910 (PMC10202454; doi:10.7554/eLife.84910)

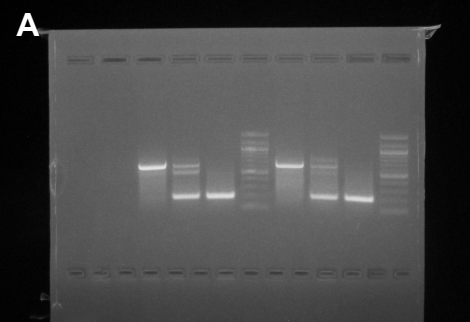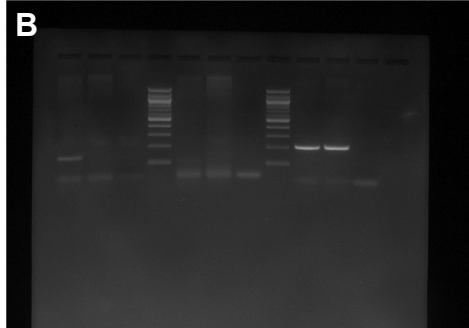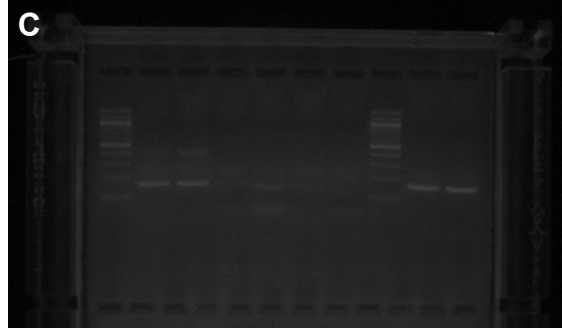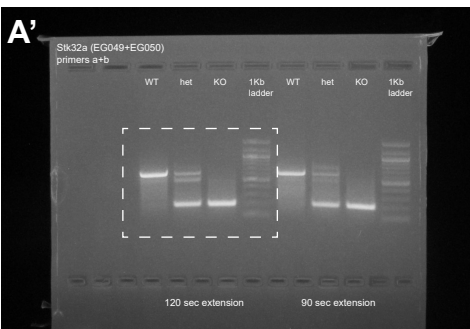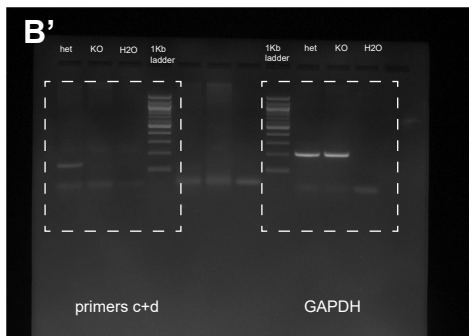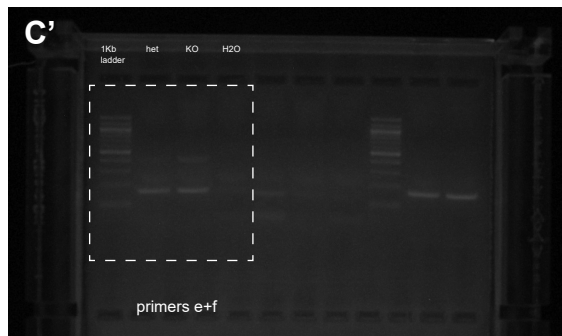

Figure 4 - figure supplement 1E - source

Figure 4 - figure supplement 1F - source

Supplement: Figure 4—figure supplement 1—source data 1. — (A) Original and (A’) annotated PCR gel demonstrating the Stk32a gene deletion generated in the Stk32a KO line. (B) Original and (B’) annotated RT-PCR gel demonstrating the absence of Stk32a coding sequence exon 2 in the Stk32a KO line and GAPDH control reactions. (C) Original and (C’) RT-PCR gel demonstrating the presence of 3’ Stk32a coding sequence exons in the Stk32a KO line. Dashed lines indicated portions of each gel that were cropped and presented in (Figure 4—figure supplement 1). Remaining lanes contain reactions that either failed or were not pertinent to that figure. [file elife-84910-fig4-figsupp1-data1.pdf]
